# The rebel, the professor, and the entrepreneur: Qualitative study to explore creator stories of FOAM

**DOI:** 10.1002/aet2.10892

**Published:** 2023-07-11

**Authors:** Matthew Zuckerman, Parnian Pardis, Tanya Horsley, Bhargavi Dhanireddy, Yusuf YIlmaz, Michael Gottlieb, Brent Thoma, Teresa M. Chan

**Affiliations:** ^1^ Department of Emergency Medicine/Division of Medical Toxicology University of Colorado, School of Medicine Aurora Colorado USA; ^2^ University of Toronto Toronto Ontario Canada; ^3^ The Royal College of Physicians and Surgeons of Canada Ottawa Ontario Canada; ^4^ University of Illinois College of Medicine Peoria Illinois USA; ^5^ McMaster University Hamilton Ontario Canada; ^6^ Ultrasound Division Rush University Medical Center Chicago Illinois USA; ^7^ Department of Emergency Medicine University of Saskatchewan Saskatoon Saskatchewan Canada; ^8^ Division of Emergency Medicine, Division of Education & Innovation McMaster University Hamilton Ontario Canada; ^9^ Department of Medicine, Department of Health Research Methodology, Evidence, and Impact McMaster University Hamilton Ontario Canada; ^10^ McMaster Education Research, Innovation, and Theory (MERIT) Program, Faculty of Health Sciences McMaster University Hamilton Hamilton Ontario Canada

**Keywords:** blog, FOAM, free open access medical education, open educational resource, podcast, social media

## Abstract

**Introduction:**

The free open access medical education (#FOAMed, or FOAM) movement creates educational content intended to inform medical education and clinical practice and is distributed in an unrestricted fashion (e.g., open access website). The who, what, and in particular the how of FOAM has raised important questions about the sustainability of the movement.

**Methods:**

We recruited a diverse research team that included educational researchers, FOAM contributors, a business academician, and medical trainees to design and conduct a qualitative study exploring the work of FOAM creators. We analyzed the transcripts of interviews with 11 participants from top FOAM websites in emergency medicine and critical care. The team met frequently to iteratively identify and discuss emergent themes (major and minor) until saturation of concepts was achieved.

**Results:**

Creators of FOAM could be categorized using three archetypes: the rebel, the professor, and the entrepreneur. The rebel was categorized as distinctly rejecting “traditional academic structures” yet was compelled to deliver educational content via alternative routes. The professor retained a traditional academic role, instead creating FOAM to supplement academic activities (teaching courses, disseminating scholarship, promotion). Entrepreneurs focused on creating a sustainable entity in an effort to supplement their income and reduce clinical obligations.

**Conclusion:**

While all FOAM creators appear unified in their passion to create, promote, and distribute educational material with unfettered access to educators, their motivations for creating content could be differentiated. Given the grassroots nature of FOAM, creators share concerns related to financing, time commitments, and threats to sustainability of these businesses. The longevity of FOAM and what business models are best suited to support them are uncertain. Further exploration of the implications could investigate the best ways to engage with and support the different FOAM creator archetypes and develop models of sustainability.

## INTRODUCTION

Free open access medical education, herein called FOAM, is a phenomenon ostensibly defined as “… a globally accessible crowd‐sourced educational adjunct providing inline (contextual) and offline (asynchronous) content to augment traditional educational principles.”^1^ At its core, FOAM is educational content that is made freely accessible via various technologies including podcasting, blogs, and social media and widely understood as being available “to anyone, anytime, anywhere.” With origins that predate the emergence of these disruptive technologies, the movement of FOAM is playing an increasingly important role in education of medical specialties.^2^, ^3^ Scholarly work has been conducted investigating the impact and quality of these online, open access, resources and suggests that they are commonly used by learners to keep up with current literature and learn core content.^1^, ^2^, ^4^

Despite our increasing reliance on FOAM resources and growing scholarship on its use and application, little is known about the FOAM motivations, particular goals, and experiences of individuals who functioned as creators of the resources. Addressing this gap is vital to sustaining an essential source of information. For those within academia, a better understanding of what motivates FOAM creators may provide transferable insights and wisdom that can be applied more broadly to other scholarly pursuits.^5^, ^6^, ^7^, ^8^ For those who seek to become FOAM creators, it may serve as a blueprint to success in FOAM and barriers to expect along the way. For consumers, the origin stories, motivations, and insights could influence how they engage with content (e.g., critical appraisal, financial support). FOAM sites, like all organizations, are often a reflection of those who create them; understanding the people who lie at the heart of the FOAM movement may provide critical insights into the origins of FOAM and a peek into future directions of this phenomenon. We conducted a qualitative, interview‐based study of individuals who were identified as creators of FOAM to gain a rich understanding of their motivations and perspectives that have shaped their experiences and work.

## METHODS

### Research paradigm

In this qualitative research study grounded in the constructivist research paradigm, the aim was to analyze the motivations of FOAM creators through in‐depth interviews. We acknowledged that knowledge and reality are constructed through participants’ experiences and social interactions. We interviewed the leaders of FOAM websites to identify underlying themes and phenomena guiding their entry and continuation into FOAM. Our inductive, interpretive description methodology thematically explored the motivations and experiences of our participants.^9^

### Research team

Our research team included faculty with clinical (MZ, MG, BT, TMC), nonclinical medical (YY), medical students (PP, BD), and business expertise (TH). Half of the researchers identified as female and half as male. Three of the researchers (MZ, TMC, BT) had prior relationships with several participants and have been heavily involved in the production of FOAM resources. Two of the researchers (TMC, YY) had formal training in qualitative methods.

### Participants

The site administrators of the top 25 emergency medicine and critical care FOAM websites as indicated by the Social Media Index were identified as eligible participants.^10^, ^11^, ^12^ They were contacted via email or their website and invited to participate in a semistructured interview regarding their role as creators. Participants were informed a priori that the purpose of the study was to determine the value of their sites as well as the inputs required to produce these sites.

### Ethics approval and reporting standards

Ethics approval was sought through the Hamilton Integrated Research Ethics Board (approval HIREB‐11529). Participants consented to being recorded. Our results are reported in compliance with the Standards for Reporting Qualitative Research checklist.^13^

### Data collection

Participants engaged in a single, virtual interview session with one of four members of the research team (PP, MZ, BD, TMC). All interviewers were trained by the senior author. Semistructured interview prompts were constructed into the interview guide (Appendix S1). Interview prompts were pilot tested with three exemplar participants. Participants did not review transcripts following their synthesis but did agree to respond via email if clarification was required. All interviews were conducted and recorded using Zoom (Zoom Video Communications, Inc.). Audio files were transcribed by a trained medical transcriptionist. Transcripts were anonymized and identifying details were redacted. Transcripts were verified or corrected if required by the analysis team.

### Data analysis

Five authors (MZ, PP, TH, BD, YY) conducted the analysis. The remaining authors reviewed and checked the source data against the analysis via an internal auditing process.

The qualitative data were analyzed by the researchers using thematic analysis to determine the routes of entry for, resources available to, and barriers faced by FOAM creators. The authors coded two interviews line by line to develop an initial list of codes and to develop an inductive coding framework. This framework was then used to code the next transcript. This continued until all of the transcripts were coded. Coding discrepancies were resolved by discussion until consensus was reached. The research team met multiple times over 6 months to analyze the codes and framework. New codes emerging during coding were added to the list upon consultation with the research team. The lists of codes were regrouped into larger categories as connections emerged from the data. Versions of the coding tree were kept in Google Docs. Themes were constructed by analyzing, combining, and comparing codes and the relationships between them. Analysis was undertaken concurrently with data collection to check for thematic saturation. This systematic approach to the analysis established an audit trail from the transcripts of raw data through to the final interpretation. Quotations, linked to respective interview sources, were presented illustrated major and minor themes.

### Images

Images for each archetype were generated by NightCafe Studio AI Art Generator with iterative prompts (e.g., “doctor with computer and headset Mark Brooks and Dan Mumford, comic book art, perfect, smooth”).

## RESULTS

Eleven of the top 25 Social Media Index site administrators, aged 35–63 years, agreed to participate in our study. Eight (73%) were male. Interviews lasted between 14 and 48 min, yielding 125 pages of transcripts. All participants were physicians. Interviewers recused themselves if there was a pre‐existing relationship with a study participant and were replaced with a different trained interviewer.

**FIGURE 1:**
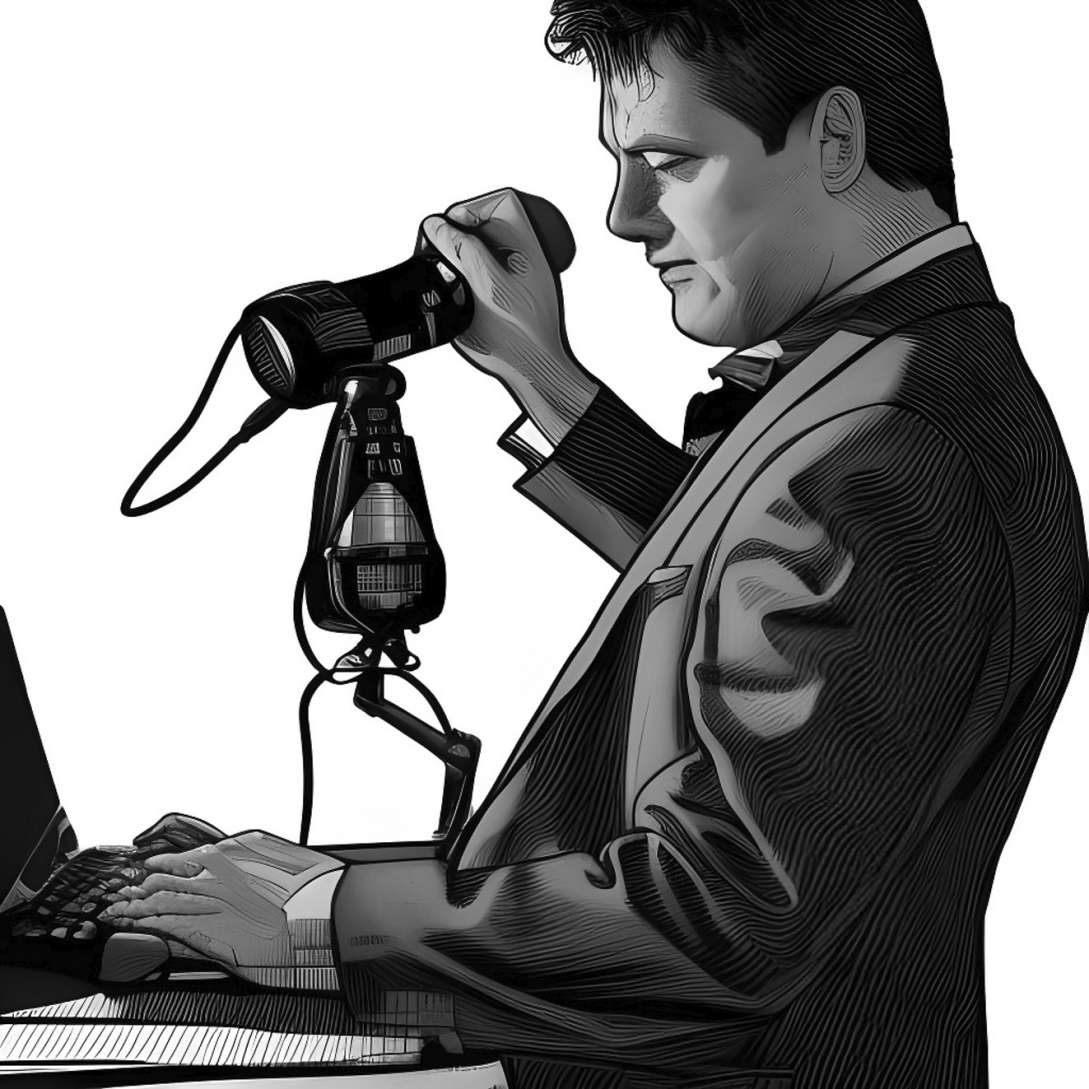
An artificial intelligence generated illustrative image of the “rebel” free open access medical education (FOAM) creator‐subtype.

**FIGURE 2:**
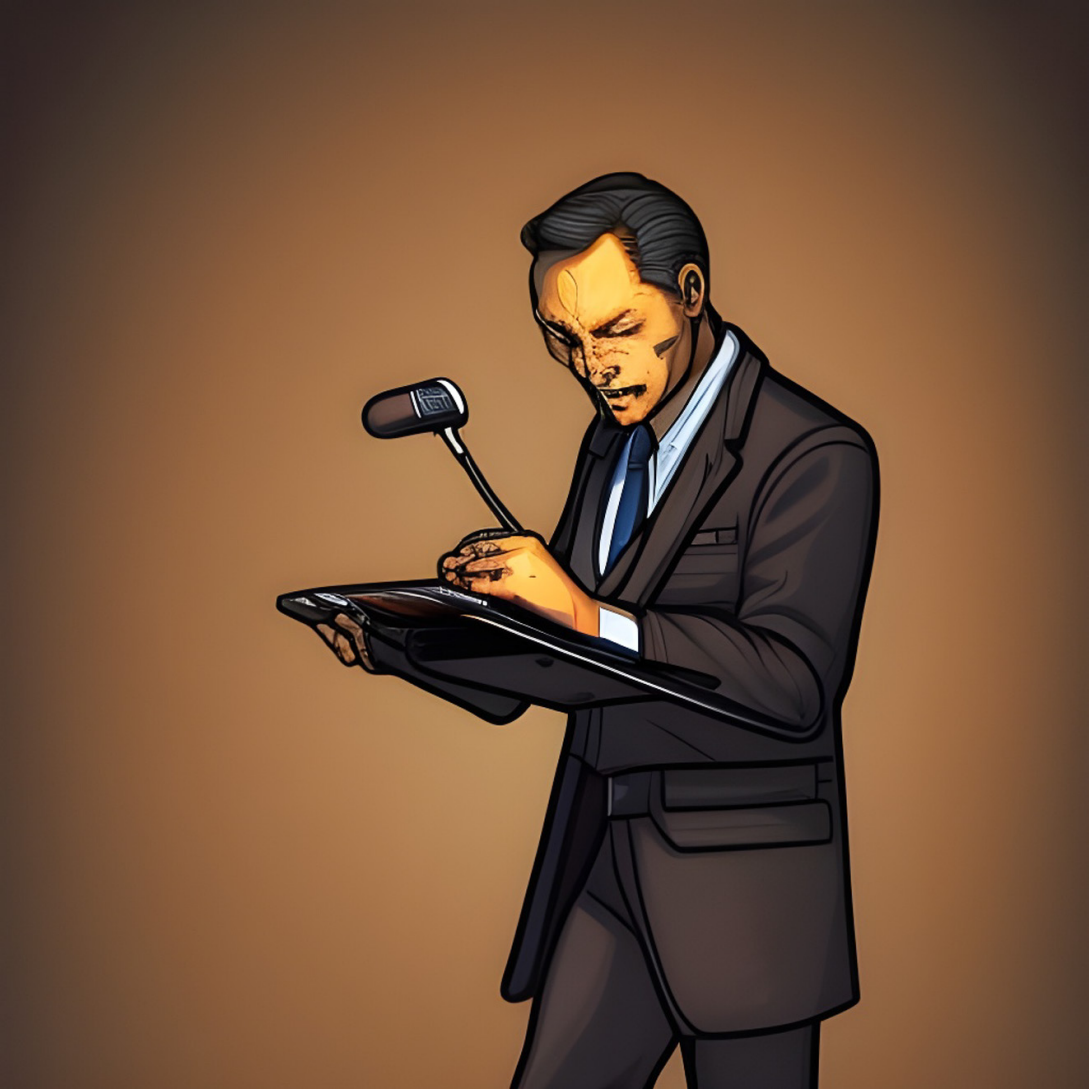
An artificial intelligence generated illustrative image of the “professor” free open access medical education (FOAM) creator‐subtype.

**FIGURE 3:**
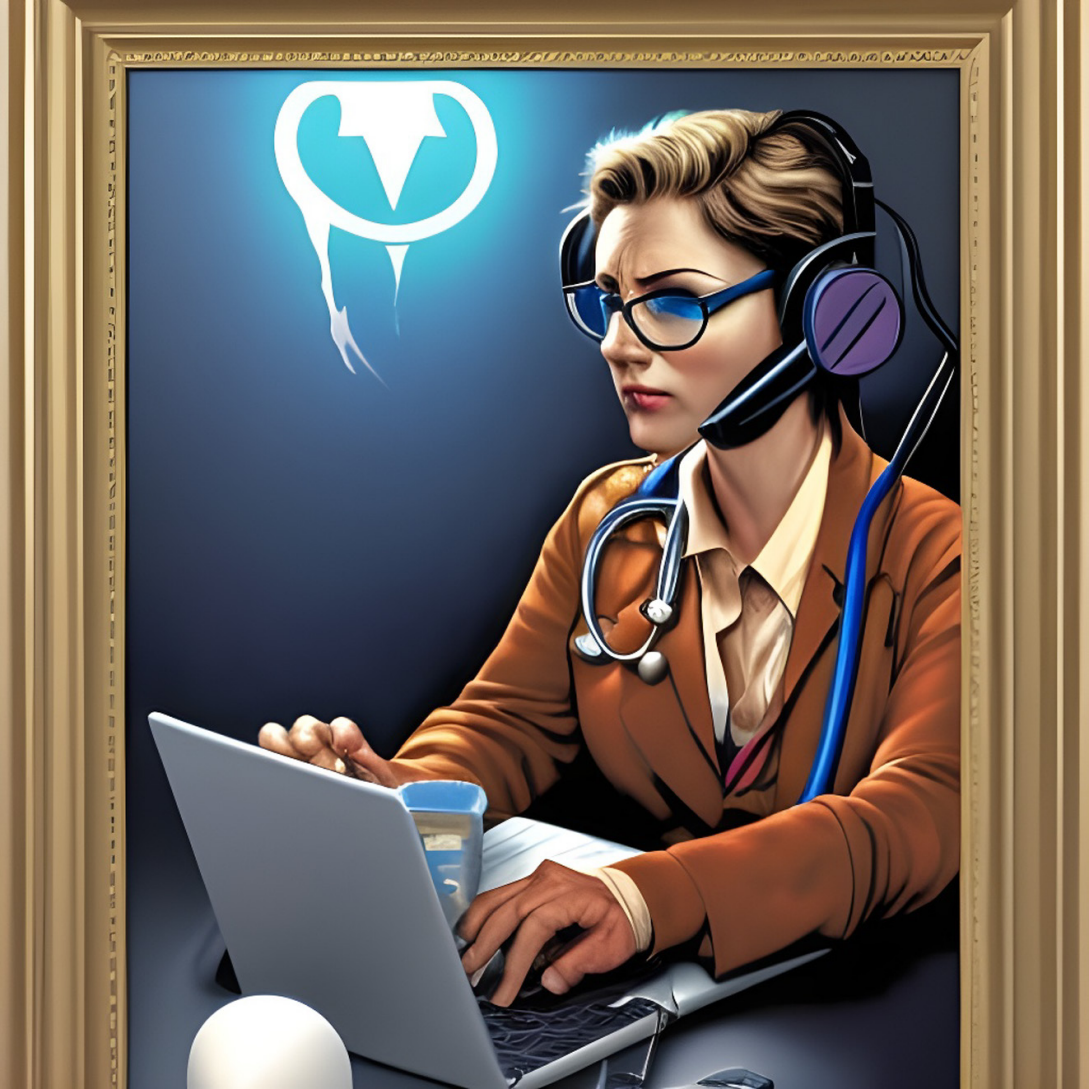
An artificial intelligence generated illustrative image of the “entrepreneur” free open access medical education (FOAM) creator‐subtype.

Our analysis identified three themes: origins, motivating factors, and resources to overcome barriers. Though origins overlapped a great deal, an analysis of motivating factors and responses to obstacles resulted in an organizational framework around three spontaneous FOAM archetypes (Figure 1, 2, 3). Major themes are summarized in Table 1 and further elucidated below.

**TABLE 1:** Major themes.

Themes	Summary
Origins	Most participants identified a common story of wanting to use new tools to solve problems, collaborate, and educate.
Motivations	This theme varied between the archetypes. Whereas rebels were motivated to continue education outside of a traditional academic institution, professors utilized FOAM to advance a traditional academic career. Entrepreneurs frequently sought to reduce clinical demands through monetization of FOAM.
Resources to overcome barriers	This theme varied between archetypes. Whereas rebels improvised to overcome barriers, professors transferred principles and resources from academia to their FOAM projects, including peer review. They also viewed FOAM as a tool to overcome barriers found in traditional course teaching (e.g., customized learning materials). Entrepeneurs focus on diversified revenue as a primary goal.

### Origin stories

While almost 90% of emergency medicine residents regularly consume FOAM resources, a small minority of them will become contributors.^4^ This led us to evaluate the origin stories of FOAM creators. What led them to write their first blogpost or record their first podcast? The FOAM mindset seemed to focus on problem solving, opportunities for collaboration, and interest in education. Participants repeatedly identified an interest in applying new tools to solve problems, especially highlighting the free distribution of educational content:

I felt that it was an opportunity to take the social media platforms which are free and open and available and ameliorate them in a way that was able to be used for medical education purposes when currently it wasn't. It was being used, which it currently is as well for cat gifs and various other things. (Participant 6)

Participants were motivated to collaborate with others and, in particular, leverage the ability to reach an expanded number of individuals in medicine. One participant highlighted this in the following quote:

And I just saw it as an awesome opportunity to actually use the platform … to get to know colleagues from around the world and to share medical information. (Participant 6)

A number of participants explained the unstructured, unplanned nature of their site's organization. Social media and websites offered yet another opportunity for engaging in conversation and at a scale unrealized in most traditional pathways of communication:

I think most of us just are classic loudmouth doctors who thought their opinions mattered about something, so they just started putting them on the internet. And people started to listen to those opinions. Um, and so it just started to grow naturally. I never had a plan to make it grow. I probably could be a lot better. I am not very good at making you know there is … search engine optimization and advertising. I don't do any of that kind of stuff. So, it just kind of naturally, though quite nice actually. (Participant 4)

Sometimes these online conversations on microblogging platforms prompted users to work together to create longer form blogs and podcasts:

We just thought, I just thought that this space for a [name redacted] blog and I just put out a tweet does anyone want to start a [name redacted] blog. And three people replied. I hadn't met them before. And, um, that was it. And we just got together. And now we are still working on it eight years later. And they are my closest friends. So, I was lucky. (Participant 5)

### The rebel: ex‐ or antiacademic

Initial innovators in FOAM were educators who had left mainstream, traditional systems of academic medicine, but wanted to engage in forms of education delivery and collaboration with colleagues. These creators were motivated to challenge the concept that an academic physician necessarily works at a university hospital and aligns with a particular faculty. As “ex‐academics” they expressed dissatisfaction with formal academia, already having transitioned out of formal, professorial roles. Despite this, they identified as educators with a desire to continue to teach and disseminate education that would improve patient care:

… And this would stand out as the fact that I had just kind of burnt out of academics at one point. And given up a lot of departmental titles. But then I didn't really have a transition point of oh, what am I going to do next. I just knew that I was an educator. (Participant 1)

Social media has been lauded for its ability to allow educators the ability to create content that can be communicated directly, without the traditional requirements imposed by journalism or traditional academic distribution outlets.^14^ This direct‐to‐consumer communication was perceived as an attractive motivator for “rebels”:

I think the reason that I went into academics was because of teaching and education and working and mentoring people. And what I found is that the further along I matriculated into the ladder of promotion and tenure and taking on different positions, the less I could teach because I was in more meetings. I was working less clinically. I had more forms and checkboxes that I had to fill. And I feel like [our] platform has been such a nice way to continue teaching, continue mentoring and doing all of the things that I am passionate about without having all of the academic checkboxes that are required when you are in academics. (Participant 2)

Though faculty may have left academic medical practice for personal reasons or as a result of frustration with academic institutions, many were still attached to some types of academic work even when they have given up formal academic roles^15^:

So, there are people who, um, can write and have an interest in academia but for whatever reason aren't pursuing an academic job but they can still engage in it. So, we've got one person in our group who is clearly heading in [a] very academic pathway and was going to be a professor of emergency medicine in the future but for personal reasons has had to step back from that. Sort of trail of paper, paper, paper. And they are a very strong supporter of what we do and still has an academic output but through slightly different means. (Participant 3)

“Rebel” archetypes frequently described a common theme of overcoming barriers in a relatively unstructured business model and a “learned as I go” mentality, frequently personally financing development. While enthusiastic about the growth of their audience, associated costs increased in parallel. Efforts to offset personal financial costs were pursued through advertisements, sponsorships, and subscriptions. However, the return on these nonacademic related pursuits failed to pay substantive dividends:

But ads on the internet just don't make any money. The people who are making money are Google, they are not the person who owns the website. So, it might be worthwhile to break even. Hell, I would love to have a second source of income that I could retire on, but it is just not going to be realistic. If you told me starting tomorrow we can give you ads that make you even half as much as a doctor, sign me up. But this ain't happening. (Participant 4)

Unlike their more traditional, academic–faculty counterparts, rebel creators had little opportunity to reduce clinical hours to compensate for their educational productivity. Clinical output was a primary metric for compensation. They were acutely aware of the negotiation needed between investment in creating FOAM materials (free labor) and how that impacted clinical work hours (paid labor) and/or their own personal time:

So, there was [cost] to that. They are obviously financial downsides because if you are doing volunteer work then you are not doing paid work at that time. (Participant 5)

I have had a relationship breakdown over the amount of time that I spend working. And so, you do 60 hours a week of normal work and then on top of that I do 40 hours a week of writing. And that can become a burden to people involved in our relationship. (Participant 6)

### The professor: traditional educator 2.0

The “professor” was the archetype whose motivation was less about rejecting traditional academia and more about taking advantage of open networks and comparatively low distribution costs to disseminate materials beyond their classroom:

It has connected me with people from all over the world. It has, which has been a seed for lots of new academic work, lots of publications. And ideas for publishable studies. And collaborators on publishable studies. (Participant 7)

I was interested in education. And working in [a] community hospital, the education role was limited to teaching you know 20 residents at a time … I started to put a few things online. And all of a sudden that 20 turned into 100 and then it was a couple million. (Participant 4)

These more traditional educators identified academic publication, promotion, and research as a major motivation for engaging in content creation. They traveled to medical conferences to speak and often published FOAM content on the same topics. They disseminated blog posts with similar content to articles published in traditional journals, but they found the impact and positive response to be markedly different. Traditional publication pathways (e.g., firewalled articles) had limited engagement with readers, relying on online discussion or traditional letters to the editor, but a perceived benefit of FOAM creators was access to immediate metrics:

But absolutely it was very different because, um, getting an article cited versus having, you know, now we are getting almost like 50,000 page views a month. And it is like: “Wow, I can reach a lot more people.” It was a lot more … thinking about interacting with people from all across the world … it was awesome. … Like I published a bunch of papers on ultrasound, but I didn't get nearly as much engagement as I do now with actual people using it. (Participant 8)

FOAM creators who remained as part of a traditional academic promotions track perceived a barrier of being defined by traditional performance metrics like publishing in academic journals. That FOAM was not yet perceived as a traditional publication pathway (e.g., limited peer review of content placed direct to social media) was a concern that they grappled with^16^:

I think at the beginning there was certainly an idea that this was poorly reviewed and opinion based anecdotal medicine. And I was involved in a number of debates around that with people like [another person] and a number of publications I have been involved in which have debated exactly that point. I think we have kind of moved beyond that. (Participant 3)

To bridge the perceived gap, several participants transferred principles of traditional academia by creating editorial processes similar to journals and textbooks for content review:

… [A]t the beginning we always had level of peer review. Within the system. But, um, it was a little bit haphazard at whereas now we have a fairly strict protocol where anything that goes in the blog would have been read by at least two of the editorial board. And only two people have the authority to press go which is me and [co‐editor]. So, the final ultimatum goes with us about how that happens. So, in the early days when it was more of a collective group of people of equal sort of rank and belonging that has sort of had to become a little bit hierarchical for quality issues. (Participant 3)

Professor archetypes described using the FOAM resources they created to augment traditional classroom and bedside educational materials. While many residents and medical students already used FOAM resources created by others, these “professors” were motivated to create their own custom images and references to reinforce their classroom approach to a particular topic:

I think the level of engagement and also one of the other motivators to make this content was to not have to teach this every single day. You know, because when a student or resident comes to me I'll be like go read this first, this is exactly how I would want to have it taught to them. Like this exact diagram that I would want to draw out or whatever. It takes a long time, it takes like three months to make a blog post. But after I am done with that I can just refer students to it. And that is a real motivation for me. (Participant 8)

Ultimately, many of these creators appeared to leverage their FOAM activities to promote traditional forms of scholarship, collaboration, and promotion in their academic environment:

I am a professor and the only reason I am a professor is because of [this] platform. So, it has definitely helped me, um, in my academic career. Um, and it is the only thing, it is really it is 99% of my academic work is [this] platform. So, it is the pretty much sole thing that has accelerated my academic career. (Participant 9)

### The pro: Entrepreneur

A third archetype creator was categorized as the “entrepreneur.” Characteristic of this creator was the motivation to create content as a sustainable business, a commodity, which could be produced and sold, outside of traditional medical education infrastructure. Whereas other creator archetypes initially appeared to take on FOAM without much attention to the resources required to produce it, entrepreneurs prominently identified the need to generate revenue streams to support their FOAM organization as a central discourse of their interviews. One participant (Participant 10) stated: “I really hope it keeps going because that will help motivate us to create more free content because we assume that the free content drive is to the paid content.”

Many creators, clinician entrepreneurs, were motivated to create FOAM as a viable alternative revenue stream as a means of reducing clinical workload:

But my main goal is to basically not have to work as many shifts so that I can spend time with my family and then still produce this content. That is actually like my goal with all of this. (Participant 10)

For others, the demands of growth soon shifted their view from traditional academics to an entrepreneurial mindset. The sheer cost of content production and web hosting made financial planning and revenue options obligatory:

Like you can start it with you just putting in a couple of hundred quid each and every so often. But five years later you know when you are getting loads of website views and you have server costs that are reasonably large. Plus, other costs you know graphics and design and coding, and it all adds up. And eventually we realized that actually if we wanted to keep growing which we did, that we would need to plan that involved some financial planning as well. (Participant 5)

While advertisements were ubiquitous for funding websites and video‐based content, participants described the sobering realities associated with such revenue streams:

I do not get ad revenue from YouTube because you have to have so many I think it's so many hours or so many followers before you get to that. I am trying to build up to that. But I haven't gotten to that yet. (Participant 2)

Some participants attempted to overcome financial barriers by utilizing a subscription model or by organizing paid conferences and research associated with their FOAM:

[We] actually started as a subscription model. And then we, that was just before FOAM sort of started taking off … We saw the advantages of FOAM, but we needed to get some sort of funding. So, we went FOAM, we went free and instead of a subscription model we have funding from a research and education institute. (Participant 9)

## DISCUSSION

This study categorized a purposive sample of FOAM content creators into three major themes and three distinct archetypes: rebels, professors, and entrepreneurs. This categorization allowed the detailed exploration of the variability and similarities in their origins, motivations, and resources to overcome barriers. Both professors and rebels are motivated to teach. Entrepreneurs are motivated to market an educational product, and they are more acutely aware of the resource costs.

FOAM creators can be differentiated but are unified in their beliefs of the benefits of FOAM in education as well as its affordances for increased networking with peers. Though it is tempting to assign these archetypes to particular historic “waves” of FOAM creation, they currently exist in parallel.^2^ Though many early FOAM producers were necessarily, “rebels,” the current state of FOAM brings more formal recognition by universities and organizations, allowing both rebels and traditional academics to participate. With FOAM established, a third type of content producer developed seeking to monetize FOAM. Similar to physician entrepreneurs in other endeavors, this group was motivated by improved lifestyle, realizing a vision, having an impact on patients outside their clinic, and solving frustrating problems.^17^ Participants varied as to whether they see these activities as unpaid academic work (professors) versus preprofitable startup (entrepreneur) versus an expensive hobby (rebel).

Rebels were heterogeneous in their reasons for operating outside of academic medical institutions. While some of them were reacting against a promotions structure that rewards meetings and non–student‐facing activities, others sought to reengage despite having to leave faculty positions for personal reasons. There is a significant overlap between the rebels and the professors. Both described the opportunity of FOAM creation to supplement or supplant traditional education, while providing opportunities for education, scholarship, and speaking. This may reflect a shift in time as FOAM gains increased recognition at academic hospitals; newer educators see the opportunity to avoid the barriers that forced ex‐academics out of academia. Key motivators among physicians who initially choose academic medicine include a desire to teach and the intellectual stimulation and challenge provided in academia.^18^

One interesting theme in the interviews is the natural way many of the participants entered FOAM. It was often organic and experimental, driven by a need to communicate beyond their current role or a way of returning to a past role. Early founders of FOAM initially disseminated work on personal websites and received little scholarly credit.^2^ Exploring the medium or teaching in a new way was frequently the initial motivation for starting to produce FOAM content. Because there were not necessarily any preexisting models of success, they were laying out a path as they followed it.

Regardless of whether they began their journey as a solo producer or part of a group, successful FOAM creators identify with a network of colleagues online allowing for growth and expansion. Some of these were “early enthusiasts” or formal members of the FOAM community of practice.^2^ The most successful creators were then able to share the burden of content creation with a group of people or ask family and friends to help out. Some of these relationships became formal in terms of research projects or hosting conferences. This evolution is seen in other industries, where a shared interest leads to networking, specialty knowledge, and the development of a formal business model.^19^

Many creators of FOAM found that success led to growth, which led to increased costs. This increased demands for time away from traditional work and from family. As the endeavor became more expensive, they frequently sought ways to monetize content to mitigate the growing burden. However, paid subscriptions and paid ads did not lead to full financial independence. While the median value of a top site can be over $23,000, this represents a fraction of the associated server, labor, and organizational costs.^20^ These factors may have contributed to a contraction in the number of FOAM sites and an increased reliance on paid models.^8^

The creators that we interviewed were similar in that many were willing to devote their own time and money to enhance medical education. This finding aligns with research suggesting that wealthier people are more likely to spend leisure time on active behaviors (e.g., exercising and volunteering) rather than passive behaviors (e.g., watching television).^21^ Physicians are often in a privileged position to engage in such endeavors, but they are not monolithic, and those with less resources may not be able to contribute funds and time to such an endeavor.^22^ For instance, women with young children have experienced a drop in academic productivity throughout the pandemic.^23^ This is also seen outside of academia where women leaders are leaving their companies at the high rates.^24^ This may also illuminate the global disparity between FOAM creation in high‐income countries versus low‐income countries.^25^

Though we identified three types of FOAM creators, they all described barriers that make continued content creation and growth challenging. This may partially explain the decrease in the number of FOAM outlets between 2013 and 2022.^8^ Perhaps some physicians will continue to be philanthropic about FOAM, freely donating their time and using their own funds to purchase equipment (e.g., recording equipment) or services (e.g., web hosting fees). Entrepreneurs may identify alternative revenue streams to mitigate cost while professors may recognize increasing benefit for their FOAM scholarly work and academic promotion. It is unclear why rebels would increase contributions as costs increase, as they do not see increasing returns on their FOAM investment.

Our collection of interviews helped define the stories and motivations of FOAM creators. They frequently saw themselves as educators more than content creators, were willing to operate outside of traditional educational settings and frameworks, and felt the burden of time and resources required for content creation. Each of them had individual reasons for engaging via FOAM, but common themes appeared as we analyzed their stories.

## LIMITATIONS

Our study is limited by a number of factors. Our enrollment strategy focused on top‐rated and currently active sites; less successful creators of smaller and defunct sites could have different stories. The lead investigators had expertise in FOAM, which may have introduced preformed interpretations. To mitigate bias from prior expertise, interviews were led and transcribed by different people and anonymized prior to analysis. To avoid potential bias resulting from the overrepresentation of minority viewpoints and expertise, we ensured analysis and reporting was performed by members of the group with disparate backgrounds and experience in FOAM.

## CONCLUSIONS

The types of free open access medical education creators—rebels, professors, and entrepreneurs—are unified in their passion to create and distribute educational material; however, they vary in experience and motivations. The study captures their motivations and highlights similarities and differences. Barriers largely come from the resources required to produce content, money, and time and can be mitigated by funding as well as recognition. Further exploration could investigate the best ways to support these archetypes. If we are to effectively foster future free open access medical education growth and sustainability, we must understand the intrinsic and extrinsic motivators for the different types of free open access medical education creators.

## AUTHOR CONTRIBUTIONS

Matthew Zuckerman, Parnian Pardis, Tanya Horsley, Yusuf YIlmaz, Michael Gottlieb, Brent Thoma, and Teresa M. Chan were involved in design, data, and interpretation; drafting and revising for intellectual content; and final approval and will stand by the work. Bhargavi Dhanireddy was involved in design, data, and interpretation.

## CONFLICT OF INTEREST STATEMENT

The authors declare no conflicts of interest.

## Supporting information


Appendix S1:
