# Supplementary material for: The rebel, the professor, and the entrepreneur: Qualitative study to explore creator stories of FOAM
Source: AEM Educ Train. 2023 Jul 11;7(4):e10892. doi: 10.1002/aet2.10892 (PMC10336018; doi:10.1002/aet2.10892)
Supplement: Supplementary file 1 — Appendix S1: [file AET2-7-e10892-s001.docx]

Hi _______. Thank you for taking time out of your busy schedule to chat with me about your (____organization_____).

So, just a little bit about myself – my name is (____name_____), and I’m a (____position____) at (____institution______). This project is being done under the approval of the XXXXX Integrated Research Ethics Board and may result in scholarly articles and abstracts. All recordings will be used for the purposes of better understanding and improving the quality of FOAM and related finances.

The interview should be about _____ minutes. The interview will be audio recorded. All data are being collected by myself and our research team. We will respect your privacy. Anyone or any institution you mention in this interview will not be disclosed. No information about who you are will be given to anyone or be published without your permission. That said, sometimes very specific incidents will make it possible to identify you, so we caution you against providing highly detailed accounts of specific encounters.

We are using the Zoom platform to collect data, which is an externally hosted cloud-based service. I have pasted a [link](https://zoom.us/privacy) to their privacy policy in the chat box. Please note that whilst this service is approved for collecting data in this study by the XXXXX Integrated Research Ethics Board, there is a small risk with any platform such as this of data that is collected on external servers falling outside the control of the research team. If you are concerned about this, we would be happy to make alternative arrangements for you to participate, perhaps via telephone. Do you consent to proceed? <participant responds>

You can stop at any time without providing a reason, and you may choose to answer only certain questions. We guarantee that your employment will not be affected by participating in this study. You may be contacted to confirm that we interpreted your responses accurately. This will be in a form of an email with key bullets of what you shared. You can clarify with us, or let us know that all the information is correct. By doing this, we are making sure that we captured your responses accurately. But if you do not wish to be contacted again after this interview, that’s fine too. So, would you agree to be contacted one more time after this interview?

<participant responds>

Great. Do you have any questions for me?

<answers questions, if any>

Do you consent to this audio recording?

<participant responds>

Great, so let’s begin! I’m turning on the recorder now.

For the record, I will begin by asking you a few questions:

1. Do you consent to be recorded?
2. Do you accept the privacy risks related to using the Zoom platform?
3. Would you agree to be contacted one more time following this interview if we require any clarification?
4. Could you please provide your age? Gender? First three letters of the city in which you were born?

We are going to start off with some questions about yourself and your role.

1. Why did you get into the FOAMed space originally?
2. What motivated you to become involved in this bigger FOAM movement as a major contributor? Or did you work pre-date this label?

*Ensure you understand motivations before you get to this

1. Considering your original motivations and intentions, do you feel you’ve achieved or realized them?
2. What are the driving forces that continue to motivate you to maintain the website?

- Have these changed over time?

1. Talk to us about your organization - <INSERT NAME OF OUTLET>. How would you describe this entity (business, hobby, scholarship/research activity, etc)? Has this changed over time?

Now we will venture into speaking about the impact of FOAM on your life.

1. How has being involved with FOAM impacted your academic or professional life?

- Pro’s: name recognition, promotion, speaking opportunities
- Con’s: unrecognized contributions, decreased academic output, negative publicity, negative feedback from peers

1. How has being involved with FOAM impacted other aspects of life?

- Pro’s: new relationships, financial
- Con’s: time away from family/friends, financial

1. Taking all of this into account, would you say that your investment has been worth it?
2. Do you have cost and revenue projections going forward?
3. Would you consider monetizing your content either through subscription or ad based revenue if you do not currently?
4. If so, would your motivation for this monetization be cost recovery? Or income? What would you spend surplus income on (e.g. reinvestment into the site / people personal income to compensate for salary, to grow a company)?
5. In the era of the pandemic, how was your organization impacted?
6. Do you have any additional comments regarding the cost/benefit of your FOAM activities?
